# Rationally Designed PU/CNFs/ZIF-8/PANI Composite Foams with Enhanced Flexibility and Capacitance for Flexible Supercapacitors

**DOI:** 10.3390/ma19071326

**Published:** 2026-03-26

**Authors:** Shanshan Li, Pengjiu Wu, Xinguo Xi, Zhiyao Ming, Changhai Liu, Wenchang Wang, Zhidong Chen

**Affiliations:** 1 School of Materials Science and Engineering, Changzhou University, Changzhou 213164, China; 2 School of Chemical and Pharmaceutical Engineering, Changzhou Vocational Institute of Engineering, Changzhou 213164, China; 3 College of Chemical Technology, Gansu Vocational University of Industry Technology, Tianshui 741025, China; 17301520919@163.com; 4 School of Materials Science and Engineering, Yancheng Institute of Technology, Yancheng 224051, China; xxg@ycit.cn

**Keywords:** polyurethane foams, cellulose nanofibers, ZIF-8, polyaniline, composite foam electrodes, hierarchical porous structure

## Abstract

Benefiting from their outstanding porosity, considerable specific surface area, and natural flexibility, cellulose nanofibers (CNFs)/MOF materials have emerged as competitive candidates for advanced flexible energy storage devices. However, conventional CNFs/MOFs aerogels or films often suffer from poor recoverability under compression, bending, and folding, accompanied by severe plastic deformation that compromises the cycling and structural stability of devices. To address this issue, we report a rationally designed flexible PU/CNFs/ZIF-8/PANI composite foam with an interconnected micro-mesoporous structure. Using polyurethane foam as a soft substrate and CNFs/ZIF-8 as building blocks, the composite was fabricated through a combined strategy of impregnation, in situ ZIF-8 growth, hot-pressing, and in situ aniline polymerization with simultaneous etching of the ZIF-8. The incorporation of carboxylated CNFs enhances the hydrophilicity of the PU skeleton. This, in combination with the hot-pressed framework, establishes an interconnected 3D network, thereby effectively preventing the agglomeration of active materials. Meanwhile, the hierarchical pores derived from the sacrificial ZIF-8 template provide abundant electroactive sites, accelerate ion transport, and facilitate high PANI loading. By virtue of this synergistic architectural effect, the resultant electrode achieves a high specific capacitance of 449 F/g at 0.2 A/g, with 97% capacitance retention after 2000 cycles at 5 A/g. Furthermore, the composite foam demonstrates excellent mechanical flexibility, with a tensile strength of 0.87 MPa and an elongation at break of 230%. This work offers a feasible approach for developing high-performance flexible supercapacitors and provides novel perspectives for the rational design of portable energy storage devices.

## 1. Introduction

The booming development of flexible and wearable electronics has raised new requirements for energy storage devices in terms of light weight, flexibility, and high stability. Thanks to the outstanding power density, rapid charge–discharge capability, and superior cycling stability, flexible supercapacitors (SCs) can adapt to complex deformations of wearable devices, including bending, stretching, and folding, ensuring a stable and continuous power supply [1,2]. As a key energy storage device for advancing the practical application of next-generation flexible electronic systems, flexible SCs hold significant research value and extensive application potential.

In the construction of flexible SCs, the flexibility of electrode materials is crucial. Recently, porous films [3], nanopapers [4], aerogels [5] and other materials based on CNFs have attracted tremendous attention thanks to their high loading capability of active materials, uniform component dispersion, and excellent flexibility and foldability. However, some CNFs-based aerogels or films exhibit poor recovery after compression, bending, and folding, and are prone to plastic deformation, which deteriorates the long-term cycling and structural stability of the devices [6,7]. Hence, it is of vital importance to explore lightweight, highly resilient flexible electrode materials. Foam-based electrodes have become a research hotspot owing to their three-dimensional interconnected network. This architecture provides outstanding flexibility and fast ion diffusion kinetics and enables high energy storage capacity when combined with electroactive materials [8,9,10,11].

Soft polyurethane (PU) foams usually possess a 3D interconnected open-pore structure, featuring light weight, good elasticity, and low cost. They have been extensively applied in daily life; typical examples include furniture cushions and vehicle seats. Recently, researchers have introduced PU foams into energy storage by utilizing their 3D porous network, mostly via high temperature carbonization [12,13,14,15]. For example, Jiang et al. [13] prepared mesoporous carbon through activation of waste PU foam and further utilized it to fabricate electric double layer capacitors, which exhibited a significantly improved energy density of 92.7 Wh/kg at 179 W/kg. Similarly, Zhou et al. [14] prepared N-doped hierarchical porous carbon (NHPC) from PU foam for SCs, and the as-obtained NHPC showed a specific capacitance of 342 F/g (133 F/cm^3^) at a current density of 0.5 A/g. However, these methods require a high temperature and inert atmosphere, leading to high energy consumption. Moreover, the carbonization process causes severe volume shrinkage and poor elasticity of the derived carbon materials, which greatly reduces material utilization efficiency.

Up to now, the intrinsic high hydrophobicity of PU has severely restricted its direct utilization as a flexible substrate for SCs, and only limited relevant studies have been reported. For instance, Wang et al. [16] employed CNFs/CNTs to modify the wettability of PU, and successfully constructed flexible SCs through a hot-pressing process. Hence, it is of great significance to explore efficient strategies to overcome the aforementioned limitations and extend the application of PU in energy storage systems.

In this paper, we present a facile fabrication route for a flexible hierarchical porous solid state SC, using soft PU as the flexible matrix and CNFs/ZIF-8 composites as the core functional components. Firstly, PU foam was immersed in a dispersion of carboxylated CNFs, followed by the in situ growth of a continuous ZIF-8 nanolayer on the CNFs surfaces. After hot-pressing treatment, the composite was subjected to the in situ polymerization of aniline under acidic conditions. Based on our previous work [17], part of ZIF-8 is synchronously etched to form defective nanoparticles during this polymerization process, which facilitates the regulation of hierarchical pore structures. Meanwhile, a nanoporous PANI coating is in situ deposited, ultimately constructing a PU/CNFs/ZIF-8/PANI (PCZP) composite foam with an interconnected micro-mesoporous hierarchical structure. The resultant composite foam can be directly fabricated into freestanding electrodes without extra binders for SCs.

In this strategy, the introduction of carboxylated CNFs effectively improves the hydrophilicity of PU foam. Combined with the hot-pressed PU framework, they form a stable and robust three-dimensional porous support structure, which provides abundant anchoring sites for ZIF-8 and PANI and prevents the agglomeration of active materials. The hierarchical porous structure constructed using ZIF-8 nanoparticles as sacrificial templates increases charge storage sites, broadens charge transport pathways, and further enhances the loading of PANI. The synergistic interaction between these components greatly promotes the comprehensive electrochemical performance of the composite foam, while endowing it with excellent flexibility and mechanical properties. This design strategy, employing PU as the flexible matrix and ZIF-8 as a sacrificial template to engineer hierarchical porous structures, not only broadens the application fields of soft PU but also provides an effective strategy for fabricating low cost, sustainable, and highly flexible SCs with superior electrochemical properties and long cycling stability.

## 2. Materials and Methods

### 2.1. Materials

Soft PU foams were collected from Nanjing Yongsheng Sponge Factory, (Nanjing, China). Sigma-Aldrich (Shanghai, China) supplied all chemical reagents utilized in this work, including sodium bromide (NaBr), monomer aniline (ANI), hydrochloric acid (HCl, 37%), ammonium persulphate (APS), 2,2,6,6-tetramethylpiperidine-1-oxyl radical (TEMPO, 98%), zinc nitrate hexahydrate (Zn(NO_3_)_2_·6H_2_O), sodium hypochlorite solution (NaClO, 6–14%), 2-methylimidazole(2-MI), and Sodium hydroxide (NaOH). The needle-bleached kraft pulp, serving as the raw material, was provided by Weifang Derui Biotechnology Co., Ltd. (Weifang, China). All reagents were of analytical grade without further purification, with the exception of aniline, which was purified via vacuum distillation before use.

### 2.2. Preparation of PU/CNFs Composite Foams

The typical TEMPO-mediated oxidation method was employed to exfoliate CNFs from needle-bleached kraft pulp, following the procedure reported in our previous work [17].

0.1 g, 0.25 g, 0.5 g, and 0.75 g of CNFs were individually weighed and dispersed in 100 mL of deionized (DI) water, followed by ultrasonication for 10–20 min to obtain uniform suspensions, respectively.

Pristine PU foam was first sectioned into 4 cm × 5 cm × 5 mm pieces. After being rinsed with DI water and ethanol and dried at 80 °C for 10 h, the pieces were impregnated with the respective CNFs suspensions described above under external force for 2 h, followed by freeze-drying.

### 2.3. Fabrication of PU/CNFs/ZIF-8 Ternary Composite Foams

The as-fabricated PU/CNFs foams were immersed in 100 mL of deionized water with constant stirring for 30 min. Two individual aqueous solutions were prepared by dissolving Zn (NO_3_)_2_·6H_2_O (0.1 g) and 2-MI (0.1 g) in 10 mL of deionized water, respectively. The Zn (NO_3_)_2_·6H_2_O solution was introduced into the PU/CNFs foam dispersion, and the resulting mixture was agitated at room temperature for 1 h. Subsequently, the 2-MI solution was gradually added into the mixture, with continuous stirring at room temperature for 1 h. The obtained products were thoroughly washed by DI water and subsequently lyophilized to afford PU/CNFs/ZIF-8 composite foams.

### 2.4. Preparation of PCZP Foams

The resultant PU/CNFs/ZIF-8 composite foams were compressed in a metal mold at 15 MPa and 150 °C for 10 min, yielding thin sheets with a thickness of 1 mm.

0.9 g aniline and 2.28 g APS were separately incorporated into 100 mL and 25 mL of 1 M HCl solution, respectively. The PU/CNFs/ZIF-8 composite foams were then soaked in the pre-cooled (0–4 °C) aniline solution for 1 h to ensure sufficient monomer adsorption. Then, the APS solution was slowly added dropwise, and the mixed solution was stirred continuously at 0–4 °C for 6 h. The products were washed successively with deionized water and ethanol, then freeze-dried to obtain PCZP foams. Based on the initial CNFs content (0.1, 0.25, 0.5, and 0.75), the resulting composites were designated as PCZP-0.1, PCZP-0.25, PCZP-0.5, and PCZP-0.75, respectively. The PANI and ZIF-8 contents in the PCZP composite foams were determined to be approximately 13.1% in weight.

To further compare the electrochemical performance of the PCZP composite foams, three control samples (PU/CNFs/PANI(PCP), PU/PANI(PP), and PU/ZIF-8/PANI(PZP)) were also fabricated. The dosages of CNFs, ZIF-8, and PANI were kept consistent with those of the PCZP-0.25 composite, and the PANI in all samples was synthesized via in situ polymerization.

### 2.5. Fabrication Flexible All-Solid-State Symmetric Supercapacitor

The flexible all-solid-state symmetric supercapacitor was fabricated by sandwiching a non-woven fabric separator (pre-impregnated with PVA/H_2_SO_4_ gel electrolyte) between two identical composite foam electrodes. The PVA/H_2_SO_4_ gel electrolyte was prepared by dissolving 6 g of PVA into 1 M H_2_SO_4_ aqueous solution at 85 °C. The composite foam electrodes were immersed in the as-prepared gel electrolyte for 30 min, and two pieces of graphite paper were employed as current collectors.

### 2.6. Electrochemical Testing and Analysis

Electrochemical measurements were carried out in a three-electrode cell containing 1 M H_2_SO_4_ as the electrolyte, with a platinum plate as the counter electrode and a saturated calomel electrode as the reference. Cyclic voltammetry (CV) and galvanostatic charge–discharge (GCD) measurements were performed on an electrochemical workstation (CHI760E, Shanghai Chenhua Instrument Co., Ltd., Shanghai, China). Additionally, electrochemical impedance spectroscopy was performed using a CHI 660D-3 instrument (Shanghai Chenhua Instrument Co., Ltd., Shanghai, China) over a frequency range from 100 kHz to 0.01 Hz, applying an AC amplitude of 5 mV.

GCD tests were performed at varying current densities (0.2–5 A/g) within a 0–0.8 V potential window, while CV measurements were conducted at scan rates of 5–100 mV/s over the same range. The working electrode had a geometric area of 1 × 1 cm^2^, electrode mass loading was measured as mg/cm^2^. The mass-specific capacitance (C*_g_*, F/g) was determined from the GCD curves according to the following formula [4]:

(1) $$C_{g}=\frac{I∆t}{m∆V}$$

Here, I (A) refers to the discharge current, Δt (s) to the discharge time, ΔV (V) to the voltage window, and m (g) to the loading mass of the active components (PANI and ZIF-8) in the working electrode.

Calculation of the mass-specific capacitance (Ccell-GCD) for the SC was performed on the basis of the GCD curves using the following formula [18]:

(2) $$C_{cell}=\frac{I∆t}{M∆V}$$

Here, I (A) refers to the discharge current, Δt (s) to the discharge time, ΔV (V) to the voltage window, and M (g) to the cumulative mass of the active components (PANI and ZIF-8) in the two electrodes.

Calculation of the energy density (E, Wh/kg) and power density (P, W/kg) for the SCs was performed using the subsequent formulas:

(3) $$E=\frac{C_{m}{\left(∆V\right)}^{2}}{2}$$

(4) $$P=3600\frac{E}{∆t}$$

In this equation, C*_m_* represents the mass-specific capacitance; energy and power densities were calculated based on the mass of active material in the device.

### 2.7. Characterization of Material Properties

The following instruments were employed for material characterization. Crystallographic information was obtained from powder XRD patterns recorded on a Bruker Focus D8 diffractometer (Cu Kα radiation, λ = 1.5418 Å) (Bruker GmbH, Karlsruhe, Germany). Functional groups were identified by FT-IR spectroscopy using a VECTOR 22 spectrometer (Bruker GmbH, Karlsruhe, Germany) over the 400–4000 cm^−1^ range at room temperature. Morphological analysis was conducted with a SUPRA-55 field emission scanning electron microscope (Zeiss, Oberkochen, Germany). XPS measurements were carried out on an ESCALAB 250 XI system (Thermo Fisher Scientific, Waltham, MA, USA) to ascertain the elemental composition. The stress–strain curves were measured using an electronic universal testing machine (ETM104B, Shenzhen Wance Testing Equipment Co., Ltd, Shenzhen, China). Additionally, a flat plate vulcanizer (D850*350, Changzhou No. 1 Rubber & Plastic Equipment, Changzhou, China) was used in the sample preparation process.

## 3. Results and Discussion

**Figure 1:**
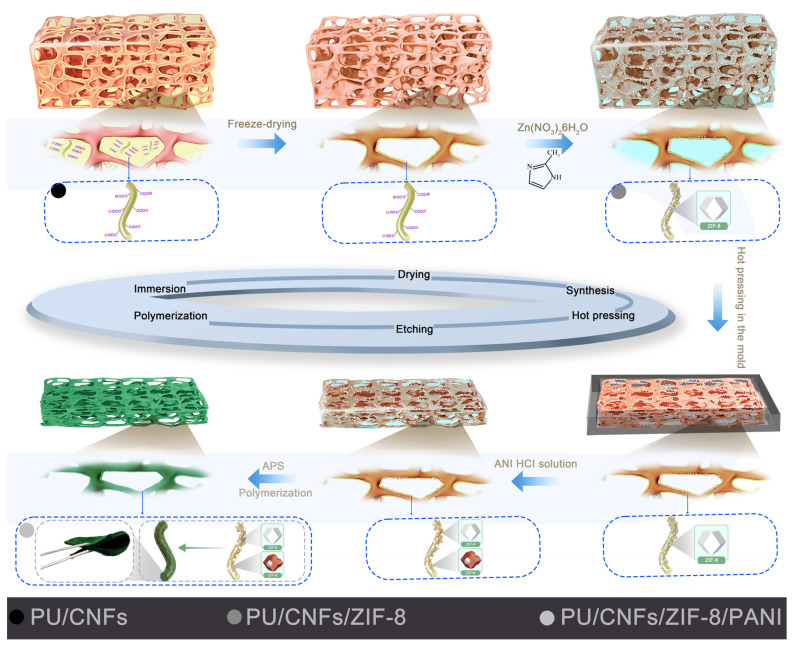
Schematic illustration of the fabrication process for PCZP composite foams.

Figure 1 schematically outlines the synthesis route for the PCZP hierarchical porous composite foams. Briefly, the CNFs obtained from softwood pulp were first subjected to TEMPO oxidation to introduce carboxyl groups onto their surfaces. Following, PU foams were immersed in CNFs suspension and then freeze-dried to obtain hydrophilic PU/CNFs composite foams with hydroxyl-rich surfaces. Subsequently, using the as-prepared composite foams as the substrate, the active sites on the surface of CNFs were utilized to induce the in situ growth of ZIF-8 nanoparticles; PU/CNFs/ZIF-8 composite foams were successfully fabricated after freeze-drying. The resultant foams were then hot-pressed into thin foams with a thickness of 1 mm, and aniline was employed as the monomer to carry out in situ polymerization to deposit PANI onto their surface. During this process, the acidic polymerization environment selectively etched ZIF-8 while retaining its porous skeleton, thereby forming a hierarchical porous structure with coexistent micropores and mesopores. Meanwhile, a nanoporous PANI coating was synchronously deposited, which infiltrated the etched ZIF-8 porous skeleton and covered the entire surface of the composite foams, constructing an interpenetrating hierarchical porous network. These as-prepared PCZP composite foams exhibited outstanding flexibility and could be bent arbitrarily into various shapes. Further details on the experimental steps can be found in the Section 2.

**Figure 2:**
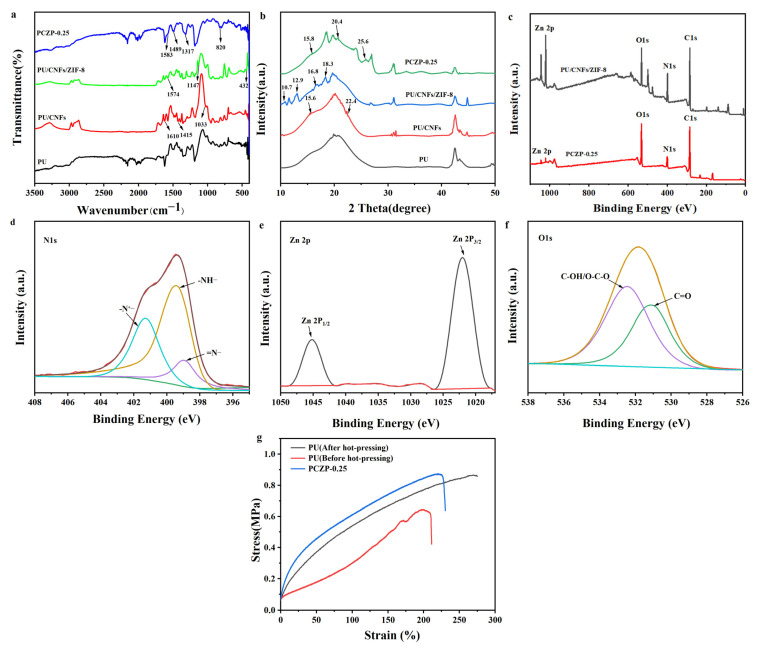
(**a**) FT-IR spectra and (**b**) XRD patterns of PU, PU/CNFs, and PCZP-0.25 foam. (**c**) XPS spectra of PCZP-0.25 and PU/CNFs/ZIF-8. (**d**) XPS spectrum of N 1s for PCZP-0.25. Colored curves correspond to deconvoluted nitrogen species: -N^+^—(cyan), -NH—(yellow), and =N—(purple). The red curve is the overall fit, the brown curve represents the smoothed line of the red fitting curve, and the green curve denotes the baseline. (**e**) XPS spectrum of Zn 2p for PCZP-0.25. The black curves represent the characteristic peaks of Zn 2P_1_/_2_ and Zn 2P_3_/_2_, respectively. The red curve denotes the baseline. (**f**) XPS spectrum of O 1s for PCZP-0.25. The colored curves correspond to deconvoluted oxygen species: C-OH/O-C-O (purple) and C=O (green). The yellow curve is the overall fit, and the cyan curve denotes the baseline. (**g**) The stress–strain curve of the thin foam.

The FT-IR spectra of PU, PU/CNFs, PU/CNFs/ZIF-8, and PCZP-0.25 are shown in Figure 2a. For the PU/CNFs composite, the peak observed at 1033 cm^−1^ is attributed to the overlapping vibrational modes of the ether groups in PU and the C-O-C bonds in CNFs [19]. Meanwhile, the peaks at 1610 cm^−1^ and 1415 cm^−1^, assigned to the asymmetric and symmetric–COO^−^ stretching vibrations, respectively, confirm the successful carboxylation of CNFs [20]. These hydrophilic carboxylate groups on the CNFs surface are anticipated to enhance interfacial adhesion with the PU matrix and improve the surface hydrophilicity of the composite foam. For the PU/CNFs/ZIF-8 composite, FT-IR spectra show characteristic peaks at 1574 cm^−1^ (C=N stretching), 1147 cm^−1^ (C-N stretching), and 432 cm^−1^ (Zn-N coordination) of ZIF-8 [21,22]. These characteristic peaks demonstrate the successful loading of ZIF-8 onto the PU/CNFs framework, while the slight shifts relative to pure ZIF-8 indicate the existence of interfacial interactions between ZIF-8 and the PU/CNFs substrate. Conductive PANI (emeraldine salt) in the PCZP-0.25 composite foam is confirmed by its characteristic peaks: 1583 cm^−1^ (quinoid C=C), 1489 cm^−1^ (benzenoid C=C), 1317 cm^−1^ (C–N^+^ stretching), and 820 cm^−1^ (C–H bending of 1,4-disubstituted rings) [23,24]. Notably, the FT-IR spectrum of the PCZP-0.25 composite foam lacks prominent peaks for ZIF-8. This can be attributed to the instability of ZIF-8 nanoparticles in the acidic environment, leading to partial structural decomposition. Furthermore, the uniform deposition of in situ polymerized PANI nanoparticles onto the composite surface contributes to the further attenuation of these signals.

The XRD patterns of PU, PU/CNFs, PU/CNFs/ZIF-8, and PCZP-0.25 are presented in Figure 2b. Compared with pure PU, two new characteristic diffraction peaks emerged at 15.6° and 22.4° for the PU/CNFs composite, which are assigned to the (110) and (200) crystal planes of cellulose I [25]. For PU/CNFs/ZIF-8, the ZIF-8 crystal structure is clearly reflected, where the characteristic diffraction peaks at 10.7°, 12.9°, 16.8° and 18.3° are in good agreement with the standard ZIF-8 pattern [26,27]. The PCZP-0.25 displayed new diffraction peaks at 15.8°, 20.4°, and 25.6° associated with amorphous PANI, indicating the successful synthesis of PANI in its doped emeraldine salt form [28,29]. Notably, the ZIF-8 characteristic diffraction peaks are not clearly visible in the XRD pattern of the PCZP-0.25 composite foam, mirroring the results obtained from FT-IR spectral analysis.

To determine the exact morphology of ZIF-8 in the PCZP-0.25 composite foam, Figure 2c compares the elemental composition and surface valence state changes in PCZP-0.25 and PU/CNFs/ZIF-8. The successful loading of ZIF-8 nanoparticles into both composite foams is confirmed by the presence of Zn 2p peaks (Figure 2c), corroborated by FT-IR and XRD analyses. The N 1s XPS spectrum with high resolution (Figure 2d) can be decomposed into three characteristic peaks at 398.9 eV (=N–), 399.4 eV (–NH–), and 401.4 eV (–N^+^–), respectively, confirming the successful introduction of PANI into the composite [30,31]. It is worth noting that the peak at 398.9 eV is located in the typical binding energy region corresponding to quinoid imine nitrogen in PANI, as well as pyridinic nitrogen from the imidazolate linkers in ZIF-8, which is likely to trigger the modulation of electronic structure. Meanwhile, two peaks are resolved in the high-resolution O 1s XPS spectrum (Figure 2f) at 531.1 eV and 532.4 eV, which are attributed to C=O and O–C–O/C–OH groups, characteristic of carboxylated CNFs [16,32]. The high-resolution Zn 2p XPS spectrum (Figure 2e) reveals two peaks at 1045.1 eV and 1021.9 eV [33], corresponding to the Zn 2p_1_/_2_ and Zn 2p_3_/_2_. Notably, a slight positive shift of approximately 0.3 eV is observed relative to pristine ZIF-8, indicating the occurrence of electron transfer from ZIF-8 to the PANI. Moreover, the concentrations of Zn and N within the PU/CNFs/ZIF-8 foam were 5.89% and 16.86%, respectively, whereas those in PCZP-0.25 drastically decreased to 0.9% and 10.3%. Consistent with our previous work [16], the acidic conditions during aniline polymerization cause etching and partial hydrolysis of ZIF-8. This process disrupts the Zn–N coordination bonds, consequently leading to a significant leaching of Zn^2+^ ions and ultimately resulting in the partial collapse of the ZIF-8 framework, and thus significant depletion of Zn and N elements. This explains the weakened Zn 2p and N 1s signals in its XPS spectrum as well as the indistinct ZIF-8 peaks in FT-IR and XRD patterns. However, the polymerization of aniline and the dissolution of ZIF-8 are governed by competing kinetics: a dense PANI coating rapidly forms on the surface of ZIF-8 particles before their complete decomposition, partially inhibiting further acid etching and retaining some intact or fragmented ZIF-8 nanoparticles embedded in the PANI matrix.

Figure 2g investigates the mechanical properties of PU foam (before and after hot pressing) and the PCZP-0.25 composite foam. The tensile strength of the composite foam was determined to be (0.87 ± 0.012) MPa, with an elongation at break measured at (230 ± 6.3%) (1.38 and 1.11 times those of uncompressed PU foam), outperforming most flexible supercapacitor electrodes based on foam substrates [34,35]. Such mechanical enhancement is due to the synergistic effects of multicomponent incorporation, structural optimization and hot-pressing treatment. The uniformly dispersed CNFs form a 3D network for stress transfer and microcrack bridging. Furthermore, hot pressing endows the composite with moderate densification, while the PU matrix maintains the flexible elastic network structure. These factors collectively contribute to the simultaneous enhancement of mechanical properties of the composite foam. The superior foldable flexibility and robust mechanical properties guarantee its practical application in flexible supercapacitors under harsh conditions.

**Figure 3:**
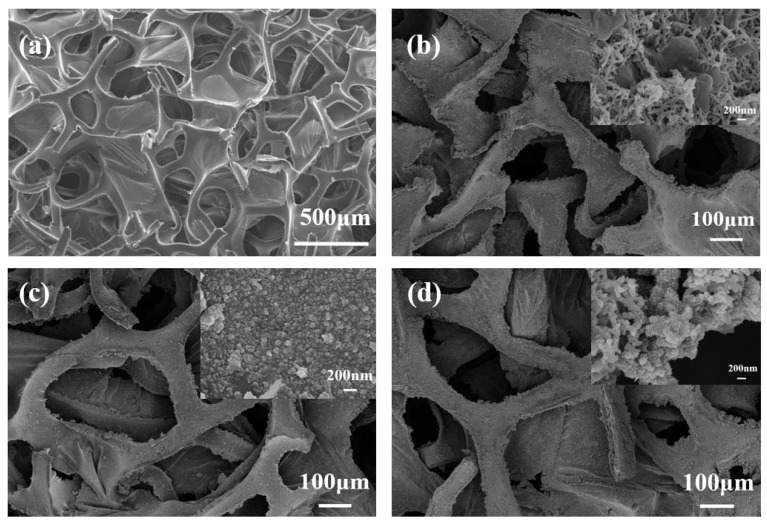
(**a**–**d**) SEM images of PU, PCP, PZP, and PCZP-0.25, respectively.

From the SEM images in Figure 3, the pristine PU foam (Figure 3a) exhibits a typical interconnected open-cell porous structure, composed of numerous irregular polygonal pores with smooth walls; adjacent pores are interconnected by thin, flexible struts to form a supporting framework. This structural characteristic—specifically, the combination of smooth pore walls and thin connecting films—largely accounts for the poor surface adhesion of most materials. After being coated with CNFs/PANI and subjected to hot-pressing treatment (Figure 3b), the PU surface turns rough, and its original porous structure is well preserved with a remarkable reduction in pore size, while the thin foam films within the pores essentially disappear. Notably, after oxidation treatment, CNFs possess a uniform nanoscale size (<100 nm), which facilitates their homogeneous dispersion within the PU matrix. This structural modification not only enhances the interfacial adhesion with polymers but also constructs abundant ion transport channels. Meanwhile, the optimized skeletal structure induced by hot pressing lays a solid structural foundation for improving the mechanical strength and electrochemical performance of the composite electrode. The PZP displays a dense and uniform particulate morphology (Figure 3c), where the surfaces and surroundings of ZIF-8 nanoparticles are uniformly coated and dispersed with PANI, with slight agglomeration observed. As clearly observed in Figure 3d, the surface of the PU framework is uniformly coated with CNFs/ZIF-8 and further covered by a loose PANI layer with abundant nanoscale pores. Specifically, the diameter of CNFs/ZIF-8/PANI anchored on the PU matrix is significantly larger than that of CNFs/PANI (Figure 3b), which is mainly attributed to the presence of ZIF-8 crystals. Moreover, the hierarchical porous structure produced by the etching process not only offers additional active sites but also provides stable anchoring sites for the efficient loading of PANI. As a result, a uniform PANI layer is immobilized on the PU/CNFs/ZIF-8 surface, which effectively facilitates the fast diffusion of electrolyte ions and promotes efficient charge transfer.

**Figure 4:**
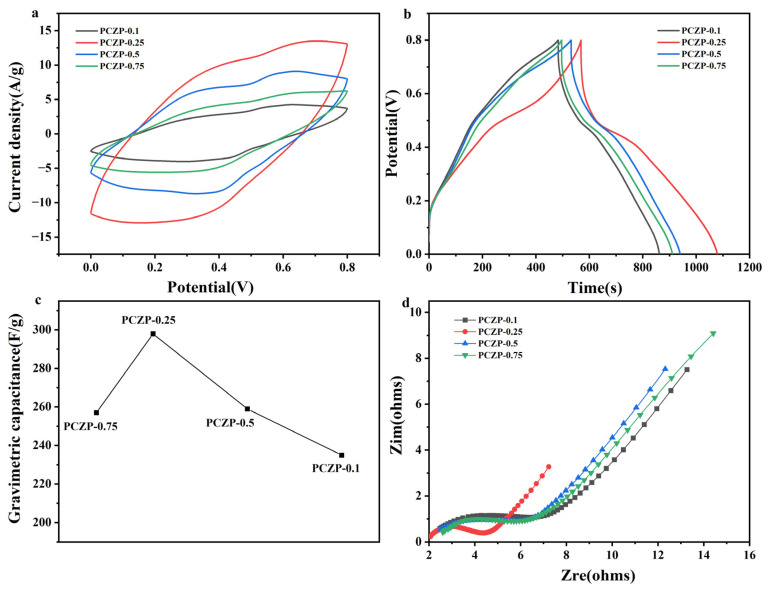
Effect of CNFs content on the electrochemical performance of PCZP foam electrodes. (**a**) CV curves at 10 mV/s. (**b**) GCD curves at 0.5 A/g. (**c**) Gravimetric capacitance at 0.5 A/g. (**d**) Nyquist impedance plots.

To further investigate the electrochemical performance of the PCZP foam, the effect of CNFs content was evaluated, with the results presented in Figure 4. As shown in Figure 4a,b, the CV and GCD curves of the electrodes with four different CNFs contents all exhibit typical redox peaks, which is mainly attributed to the reversible redox reactions of PANI [36,37]. With the increase CNFs content, both the CV area and GCD discharge time show a distinct volcano-shaped trend, first increasing and then decreasing. In the PCZP foam system, CNFs play a multifunctional role: they construct a continuous three dimensional network structure within the PU matrix, provide abundant anchoring sites for the uniform loading of ZIF-8 and PANI, effectively suppress the agglomeration of active materials, and enhance the interfacial synergy among various components. Correspondingly, at an optimal CNFs content of 0.25%, the electrode exhibits the largest CV area and longest discharge time, delivering a specific capacitance of 321 F/g at a current density of 0.5 A/g. However, when the CNFs content reached 0.75%, the specific capacitance decreased significantly to 257 F/g (Figure 4c). It was mainly attributed to the severe agglomeration of CNFs induced by overloading, which disrupts the interconnected porous structure of the composite foam. This agglomeration hinders electrolyte infiltration and ion diffusion in the bulk phase. It also reduced the effective contact area between the PANI and the electrolyte and ultimately weakened the electrochemical synergy among CNFs, ZIF-8 and PANI, resulting in a significant decline in electrochemical performance.

For a more comprehensive understanding of the electrode kinetics, EIS measurements were carried out, and the Nyquist plots are displayed in Figure 4d. The low frequency slope is maximized for the PCZP-0.25 electrode, indicating faster ion diffusion. Moreover, it displays the smallest semicircle diameter in the high-frequency region, signifying the lowest charge transfer resistance (Rct). These EIS results confirm that the optimal CNFs content (0.25%) effectively prevents agglomeration, establishing a favorable three dimensional network and interfacial structure that facilitates both charge transfer and ion transport.

**Figure 5:**
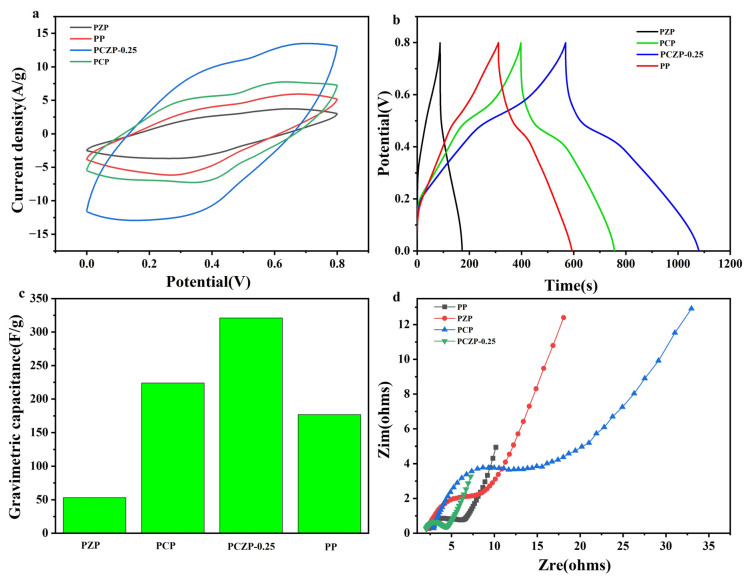
Comparison of electrochemical performance among different foam electrodes. (**a**) CV curves at 10 mV/s. (**b**) GCD curves at 0.5 A/g. (**c**) Gravimetric capacitance at 0.5 A/g. (**d**) Nyquist impedance plots.

A systematic comparison of four composite foams—PP, PCP, PZP, and PCZP-0.25—was conducted to further confirm the synergistic interactions among PU, CNFs, ZIF-8, and PANI and their collective impact on electrochemical performance, with the results presented in Figure 5. As shown in Figure 5a,b, the PCZP-0.25 electrode exhibits substantially larger CV area and longer GCD discharge time than the other three counterparts. This superior performance demonstrates that the integration of these components is more than just physical mixing or surface coating. Rather, their synergistic interplay optimizes charge transfer kinetics and minimizes ion diffusion resistance, resulting in substantially enhanced electrochemical performance. In the PZP system, the absence of CNFs meant that no continuous three dimensional network was constructed, nor were abundant anchoring sites provided for the uniform loading of ZIF-8 and PANI. This leads to blocky aggregation of ZIF-8 and PANI (Figure 3c), with an even lower specific capacitance than PP, thus yielding the lowest specific capacitance among the four systems. This comparison demonstrates the key function of CNFs in building a stable framework and realizing homogeneous dispersion of the active materials. Conversely, in the PCP system, despite the presence of CNFs, the lack of a ZIF-8 derived sacrificial template means the composite loses the hierarchical porous architecture. This porosity is critical for providing additional charge storage sites and facilitating electrolyte penetration. Accordingly, its specific capacitance is also inferior to that of the PCZP-0.25 foam, which underscores the crucial contribution of the porous structure derived from ZIF-8.

The Nyquist plots (Figure 5d) further confirm these results. The PCZP-0.25 electrode displays the smallest high-frequency semicircle (lowest charge transfer resistance) and the steepest low-frequency line (most favorable ion diffusion kinetics), providing direct evidence for the synergistic enhancement of charge and ion transport dynamics achieved through the full integration of all components.

**Figure 6:**
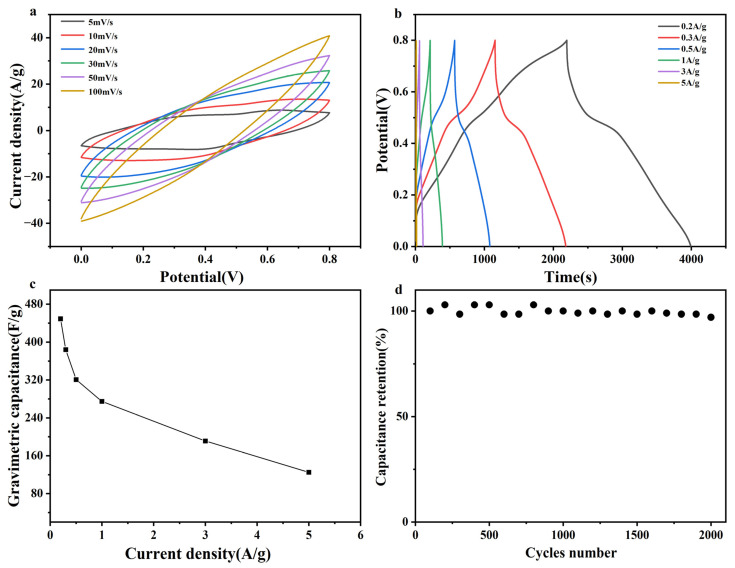
Electrochemical performance of the PCZP-0.25 foam electrode. (**a**) CV curves at scan rates from 5 to 100 mV/s. (**b**) GCD curves at current densities ranging from 0.2 to 5 A/g. (**c**) Corresponding specific capacitance values. Squares represent the specific capacitance values at different current densities, and lines represent the trend of the corresponding data points. (**d**) Cycling performance at 5 A/g.

On the basis of the preceding analysis, a more comprehensive investigation was conducted on the electrochemical performance of the PCZP-0.25 foam electrode, with the corresponding results illustrated in Figure 6. The CV curves at various scan rates (5–100 mV/s) are presented in Figure 6a. The current response increases proportionally with the scan rate, and the curve maintains a regular shape, exhibiting typical pseudocapacitive characteristics and outstanding reversibility. The GCD curves at various current densities (0.2–5 A/g) are illustrated in Figure 6b. Notably, the plateaus observed between 0.4 and 0.6 V can be ascribed to the redox reactions of PANI. As depicted in Figure 6c, the specific capacitance gradually decreases with increasing current density, reaching 449 F/g and 321 F/g at 0.2 A/g and 0.5 A/g, respectively (71.5% retention). Such a moderate capacitance decay indicates fast ion diffusion and excellent rate capability.

This performance is significantly superior to that reported in most similar literatures, such as 226.8 F/g for CNFs/CNT/PANI at 0.4 A/g and 276 F/g for Cu-MOF/rGO/PANI at 0.5 A/g. The detailed comparison is presented in Table 1.

It is well recognized that pure PANI electrodes often suffer from severe volume changes during charge/discharge cycles, leading to poor cycling stability. To assess this critical aspect, the long-term cycling performance of the PCZP-0.25 electrode was evaluated at 5 A/g for 2000 cycles (Figure 6d). Remarkably, the capacitance retention reaches 97%, which is substantially higher than most reported PANI-based electrodes [40,41,47,48]. This excellent stability arises from the robust three dimensional network constructed by the CNFs/ZIF-8 framework, which effectively alleviates the volume expansion of PANI during cycling and avoids structural collapse, and maintains stable electrochemical performance.

**Figure 7:**
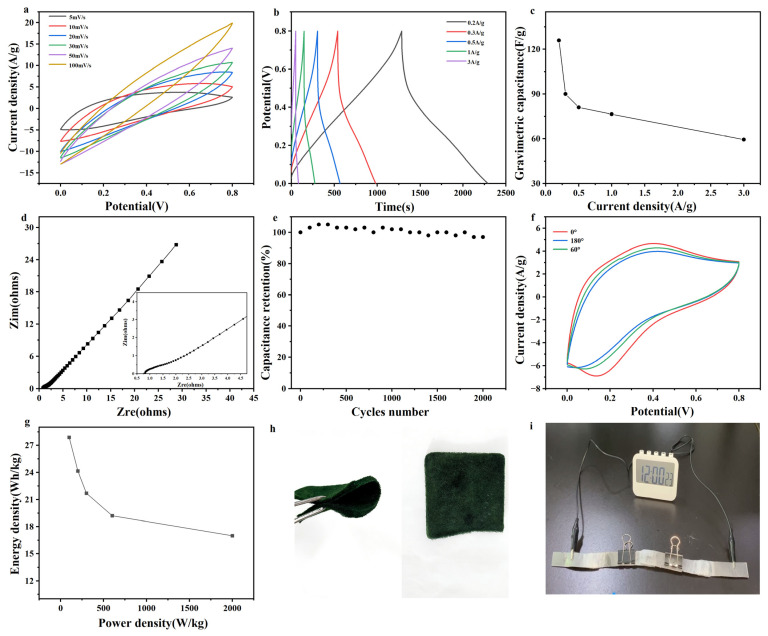
The electrochemical performance of the PCZP-0.25 all-solid-state symmetric supercapacitor. (**a**) CV curves at scan rates from 5 to 100 mV/s. (**b**) GCD curves at current densities ranging from 0.2 to 3 A/g. (**c**) Corresponding specific capacitance values. Circles represent the specific capacitance of the device at different current densities, and line represent the trend of the corresponding data points. (**d**) Nyquist plots. Squares in the main plot represent the complex impedance at specific frequencies, while those in the inset correspond to the magnified complex impedance data of the same device, and lines represent the trend of the corresponding data points. (**e**) Cycling performance at 5 A/g. (**f**) CV curve at 10 mV/s with various folding angles. (**g**) Ragone plot for the SC at various current densities (0.2–3 A/g). Squares represent the energy and power densities obtained at specific discharge currents, and lines represent the trend of the corresponding data points. (**h**) Photo of PCZP-0.25 composite foam. (**i**) Photo of timer by the devices.

Given its excellent electrochemical performance, the practical application potential of the PCZP-0.25 electrode was evaluated by assembling a flexible all-solid-state symmetric supercapacitor with dimensions of 1 cm × 1 cm × 1 mm. The device comprised two identical electrodes separated by a non-woven fabric membrane, with graphite paper serving as the current collector and a PVA-H_2_SO_4_ gel acting as the solid state electrolyte. As anticipated, the assembled SC exhibits well defined redox peaks in both CV and GCD curves (Figure 7a,b), confirming the typical pseudocapacitive characteristics of PANI.

The as fabricated all-solid-state SC exhibits a specific capacitance of 90 F/g at 0.3 A/g and retains 76.5 F/g at 1 A/g (Figure 7c), corresponding to an outstanding capacitance retention of 85.4% and demonstrating excellent rate capability. The Nyquist plot (Figure 7d) reveals an extremely low ohmic resistance (~0.78 Ω) and negligible charge transfer resistance, indicating facile ion penetration and efficient charge transfer within the porous foam structure. This advantageous kinetic behavior is attributed to the well-integrated structural design. The three dimensional network structure constructed by CNFs and flexible PU endows the electrode with favorable structural stability and provides a framework for the rapid electron transfer in PANI. Hot pressing treatment effectively shortens the ion transport pathways. The hierarchical porous structure derived from the ZIF-8 template offers rapid ion diffusion channels. The robust interfacial interaction between PANI and the substrate minimizes the charge transfer resistance and ensures structural stability during cycling.

Beyond the aforementioned electrochemical properties, the cyclic stability, mechanical flexibility, and practical application potential of the all-solid-state symmetric SC were comprehensively investigated. The PCZP-0.25 foam itself exhibits considerable flexibility, allowing it to be easily bent without structural damage and directly processed into electrodes without any additional binders or conductive agents (Figure 7h). This excellent flexibility translates to the device level: the CV curves display only minimal changes upon bending at 60° or folding at 180° (Figure 7f), confirming the outstanding mechanical stability of the flexible energy storage device. Cycling stability tests were performed at 5 A/g. As shown in Figure 7e, the all-solid-state supercapacitor retains 97% of its initial capacitance after 2000 cycles, demonstrating outstanding long term electrochemical stability.

To demonstrate practical applicability, two all-solid-state supercapacitors assembled in series successfully powered a timer for more than one minute (Figure 7i, Video S1), demonstrating the promising practical application potential of the fabricated device. Furthermore, the energy and power densities of the supercapacitor were determined and plotted in the Ragone plot (Figure 7g). At a power density of 200 W/kg, the as-prepared device achieves a high energy density of 24.1 Wh/kg and still retains 19.2 Wh/kg even under a higher power density of 600 W/kg. This comprehensive performance surpasses that of numerous previously reported conducting polymer-based SCs [39,43,47,48].

## 4. Conclusions

In summary, using PU foam as a three dimensional porous flexible substrate, a PU/CNFs/ZIF-8/PANI (PCZP) composite foam electrode with a hierarchical micro-/mesoporous structure was successfully fabricated via a stepwise strategy involving hydrophilic modification, in-situ growth of active components, structural densification, and conductive layer coating. The synergistic mechanism of each component and the structure performance relationship were systematically elucidated. The main conclusions are as follows:

(1) An integrated scheme for the hydrophilic modification of PU foam and the anchoring of active components was proposed. Carboxylated CNFs were loaded onto the surface of the PU skeleton by impregnation, which not only significantly improved the hydrophobicity of PU foam and laid a foundation for electrolyte wetting and ion transport, but also guided the uniform in-situ growth of ZIF-8 nanoparticles via abundant active sites on the CNF surface. A three dimensional interconnected “PU-CNFs-ZIF-8” supporting structure was formed, solving the problems of uneven loading of active components and weak interfacial bonding in traditional PU-based electrodes.
(2) A hierarchical pore synergistic transport network was constructed to optimize the matching between electrode structure and performance. Hot pressing reduced the thickness of the composite foam to 1 mm, which not only enhanced the mechanical stability of the skeleton but also shortened the ion transport pathways. ZIF-8 was selectively etched in the acidic environment of aniline polymerization, forming a hierarchical porous structure with interconnected micro- and mesopores. This provided abundant active sites for the uniform loading of PANI and effectively inhibited PANI agglomeration.
(3) The composite foam electrode exhibited excellent comprehensive performance. The optimally prepared PU/CNFs(0.25)/ZIF-8/PANI composite foam possessed outstanding mechanical flexibility and electrochemical performance. The single electrode delivered a high specific capacitance of 449 F/g at 0.2 A/g. The all-solid-state symmetric supercapacitor assembled with this electrode achieved a specific capacitance of 252.5 F/g at the same current density. Moreover, the capacitance retention remained as high as 97% after 2000 charge–discharge cycles at a high current density of 5 A/g, showing superior cycling stability to most similar flexible electrodes.

This work provides a novel and feasible strategy for fabricating high performance flexible energy storage devices, with the additional advantage of potentially utilizing waste polymer foam as a resource. The excellent comprehensive performance of the material highlights its great potential for applications in wearable and portable electronics, offering new insights for the sustainable development of next generation flexible energy storage systems.

## Figures and Tables

**Table 1:** Electrochemical properties of different electrode materials.

Samples	Electrolyte	Specific Capacitance for Electrode (F/g)	Capacitance Retention (Cycle Number)	Reference
CNFs/CCS/PANI	1 M H_2_SO_4_	220 (1 mA cm^−2^)	84.1% (3000)	[38]
NC/PANI/RGO-5	1 M H_2_SO_4_	79.71 (0.1 A/g)	-	[39]
CNFs/CNT/PANI	6 M KOH	226.8 (0.4 A/g)	90% (1000)	[40]
PANI/CNC	1 M H_2_SO_4_	138.12 (0.4 A/g)	85% (2000)	[41]
PANI-CNT	1 M KOH	163 (1 A/g)	-	[42]
PANI-MNW	1 M KOH	256 (1 A/g)	-	[43]
Zn-MOF/rGO/PANI	1 M H_2_SO_4_	372 (0.1 A/g)	77.18% (5000)	[44]
Cu-MOF/rGO/PANI	1 M H_2_SO_4_	276 (0.5 A/g)	82.96% (1500)	[45]
PANI/PCMs	1 M H_2_SO_4_	242 (1 A/g)	-	[46]
PCZP-0.25	1 M H_2_SO_4_	321 (0.5 A/g)	97% (2000)	This work

## Data Availability

The original contributions presented in this study are included in the article. Further inquiries can be directed to the corresponding author.

## Supplementary Materials

The following supporting information can be downloaded at: https://www.mdpi.com/article/10.3390/ma19071326/s1, Video S1: Video of the supercapacitor-driven timer.
